# Evaluating the Performance of Fine-Mapping Strategies at Common Variant GWAS Loci

**DOI:** 10.1371/journal.pgen.1005535

**Published:** 2015-09-25

**Authors:** Martijn van de Bunt, Adrian Cortes, IGAS Consortium, Matthew A. Brown, Andrew P. Morris, Mark I. McCarthy

**Affiliations:** 1 Wellcome Trust Centre for Human Genetics, University of Oxford, Oxford, United Kingdom; 2 Oxford Centre for Diabetes, Endocrinology & Metabolism, University of Oxford, Oxford, United Kingdom; 3 University of Queensland Diamantina Institute, Translational Research Institute, Brisbane, Queensland, Australia; 4 Department of Biostatistics, University of Liverpool, Liverpool, United Kingdom; 5 Oxford NIHR Biomedical Research Centre, Churchill Hospital, Oxford, United Kingdom; National University of Singapore, SINGAPORE

## Abstract

The growing availability of high-quality genomic annotation has increased the potential for mechanistic insights when the specific variants driving common genome-wide association signals are accurately localized. A range of fine-mapping strategies have been advocated, and specific successes reported, but the overall performance of such approaches, in the face of the extensive linkage disequilibrium that characterizes the human genome, is not well understood. Using simulations based on sequence data from the 1000 Genomes Project, we quantify the extent to which fine-mapping, here conducted using an approximate Bayesian approach, can be expected to lead to useful improvements in causal variant localization. We show that resolution is highly variable between loci, and that performance is severely degraded as the statistical power to detect association is reduced. We confirm that, where causal variants are shared between ancestry groups, further improvements in performance can be obtained in a trans-ethnic fine-mapping design. Finally, using empirical data from a recently published genome-wide association study for ankylosing spondylitis, we provide empirical confirmation of the behaviour of the approximate Bayesian approach and demonstrate that seven of twenty-six loci can be fine-mapped to fewer than ten variants.

## Author Summary

Over the last few years, several approaches for fine-mapping genome-wide association studies (GWAS) loci have been proposed and used to localize potential causal variants. However, the performance of these types of tests is often poorly characterized. In this study, we used extensive simulations to show that statistical fine-mapping can indeed accurately reduce the number of likely causal variants at common GWAS loci. These approaches can be further improved by changes in study design, such as the inclusion of multiple ethnic groups in the study population. Finally, we demonstrate the utility of this type of approach on a recently published genome-wide association study for ankylosing spondylitis, where we could fine-map seven of the twenty-six loci to a number of variants (n = 10) which is tractable for follow-up in a laboratory setting.

## Introduction

As a result of linkage disequilibrium (LD), loci identified by common variant genome-wide association analyses are often large and may contain hundreds of variants. Characterization of the specific variants driving these signals has clear benefits, most obviously through highlighting the specific causal molecular mechanisms, which may provide insight into complex disease pathophysiology. Progress in this endeavor has been hampered by incomplete coverage of human sequence variation (so potential causal variants are missing from genome-wide association study [GWAS] and imputation data sets), and by lingering uncertainties concerning the frequency spectrum of the underlying risk-alleles [1–3]. However, whole-genome sequence data (e.g. from the 1000 Genomes Project) now offer near complete coverage across the common and low frequency allele ranges at least (minor allele frequency [MAF] >1%) in multiple ancestry groups [4]. At the same time, evidence is accumulating—from large-scale GWAS [3,5], fine-mapping [5–7], re-sequencing [2,8] and trans-ethnic studies [9,10]–that most (though not all) GWAS signals are driven by causal variants that are themselves common, with evidence supporting alternative rare variant association models (e.g. synthetic association [1]) restricted to a few loci (e.g. *NOD2/CARD15* in inflammatory bowel disease [8]).

These advances make it possible to perform systematic fine-mapping studies that interrogate the vast majority of potential causal variants at GWAS loci. Indeed, several recent studies have reported successful refinement of GWAS loci [6,7]. These efforts often include a trans-ethnic component that seeks to leverage ancestral differences in LD patterns between common SNPs to aid causal variant localization [9–11]. However, substantive questions related to the reliability, precision and performance of GWAS fine-mapping efforts remain unanswered. In this study we combined simulation-based and empirical approaches to address these questions.

## Results

### Evaluating fine-mapping performance using approximate Bayesian refinement

To quantify fine-mapping precision, we simulated ten association studies of 1,000 cases and 1,000 controls in 1Mb regions from the CEU haplotypes present in the 1000 Genomes Phase 1 dataset [4]. In each region we assigned a single, additive effect, causal variant of varying odds ratio (OR) and risk allele frequency (RAF). For each combination of OR and RAF, we simulated 1000 random “replicate” regions. On these data, we performed case-control association testing under an additive model, followed by meta-analysis of all ten studies for each region (such that the total sample size examined was 20,000). On the basis of the meta-analysis summary statistics for each of the variants, we generated approximate Bayes’ factors (ABFs)–describing the evidence in favor of association for a given variant–using the method proposed by Wakefield [12]. From these ABFs, the posterior probability of each variant to be causal was derived, and used to assemble “credible sets” that contained all variants with a cumulative posterior probability of a causal variant exceeding a chosen threshold (here, 95% or 99%) for each of the 1000 replicate regions [6].

The haplotypes used for the simulations are derived from whole-genome sequencing data for the 1000 Genomes project [4], and as such represent a “gold standard” scenario with nearly the entire low-frequency and common variant spectrum covered. Although whole-genome sequence data are likely to offer the most accurate framework for fine-mapping, currently available whole-genome sequencing datasets are relatively small and lack power to discriminate between highly-correlated variants. As a result, most fine-mapping studies rely on the much larger datasets available from the meta-analysis of GWAS and/or custom array datasets after imputation. To represent such designs, we filtered these whole-genome data to those present on the Illumina HumanOmni2.5 BeadChip array, corresponding to genotype data equivalent to those from dense GWAS or a custom fine mapping array such as the Metabochip [13]. For this “GWAS” scenario, these variants were subsequently imputed up to the 1000 Genomes Phase 1, all ancestries, release 3 panel. Additionally, we created a “GWAS with failure” scenario–representing a GWAS with a genotype missingness rate per cohort–where, for each of the twenty case or control cohorts (each with n = 1000) in an analysis, we removed all genotypes from 5% of the variants (after downsampling). This equates therefore to an ~0.25% missingness rate per variant across each set of 20,000 samples.

From the simulated data, we first sought to establish whether the credible sets generated from these simulations by the approximate Bayesian approach had appropriate coverage of the simulated causal variant under a model of association. In the gold standard scenario (i.e. unfiltered 1000 Genomes data), where the causal variant would always be present in the sample, we estimated the median coverage of the causal variant at 98.5% and 99.8%, for the 95% and 99% credible sets, respectively (S1 Fig). In the downsampled GWAS scenario, coverage of the causal variant was broadly comparable to that under the gold standard – 97.8% for the 95% credible set and 99.6% for the 99% credible set. The introduction of a random 5% variant drop-out per study population in the GWAS with failure scenario could result in loss from analysis, in some of the datasets, of the causal variant, or of variants that are influential with respect to the imputation scaffold. ABFs were rescaled by maximum effective sample size to compensate for genotype missingness. Results showed that the introduction of random missingness primarily affected simulations with little power to detect genome-wide significant association. At all other simulated OR and RAF combinations, missing genotype data led to only modest attrition of the causal variant coverage (S1 Fig).

**Fig 1:**
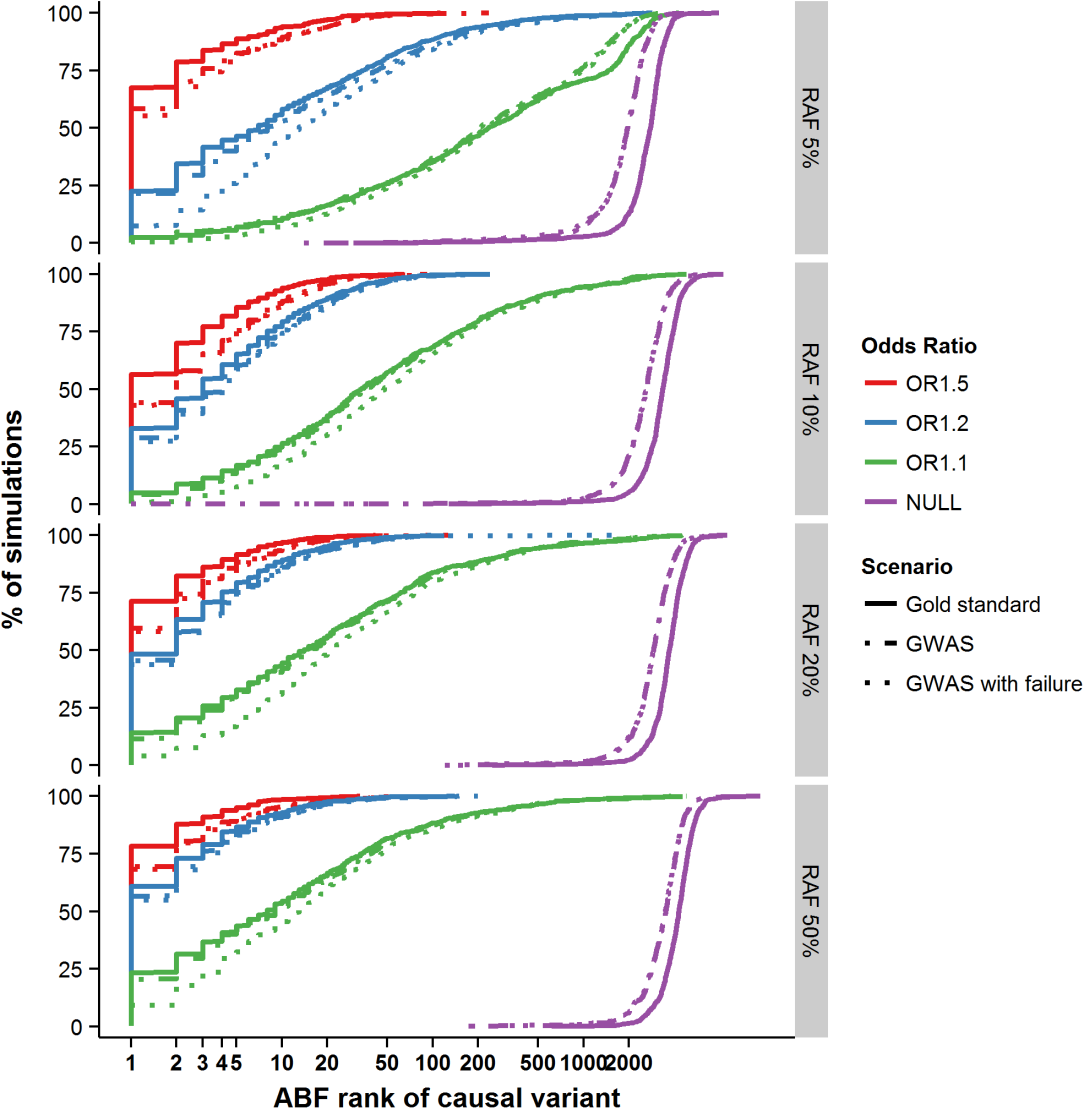
Ranking of the causal variant across simulated loci. Cumulative percentage of simulations (y-axis) with decreasing ranking of the causal variant amongst all variants in the regions (x-axis). Panels are split by risk allele frequency of the causal variant along the vertical axis.

Having shown that the credible set performance was well calibrated, we next asked how often the causal variant was identical to the most strongly associated (lead) SNP. In situations where the two show near complete overlap, more sophisticated fine-mapping strategies would be unnecessary. In the gold standard scenario where the causal variant had the highest OR (1.5) and RAF (50%), 79% of the simulations accurately identified the causal variant as the most strongly associated (Fig 1). However, when the risk allele was less common (RAF 5%) and the phenotypic effect more in line with that observed at more typical GWAS loci (OR 1.1), the probability of accurately identifying the causal variant by focusing on the most associated variant dropped to just 2.4% (Fig 1). All subsequently presented results are based on the GWAS scenario, as this most closely resembles the majority of contemporary fine mapping studies. In this scenario, the numbers ranged from 70% (RAF 50% and OR 1.5) to 2.4% (RAF 5% and OR 1.1). These results confirm that focusing solely on the most significant variant is insufficient for identification of the causal variant at most common variant GWAS loci, so more elaborate approaches to fine-mapping–such as construction of credible sets–are needed.

**Fig 2:**
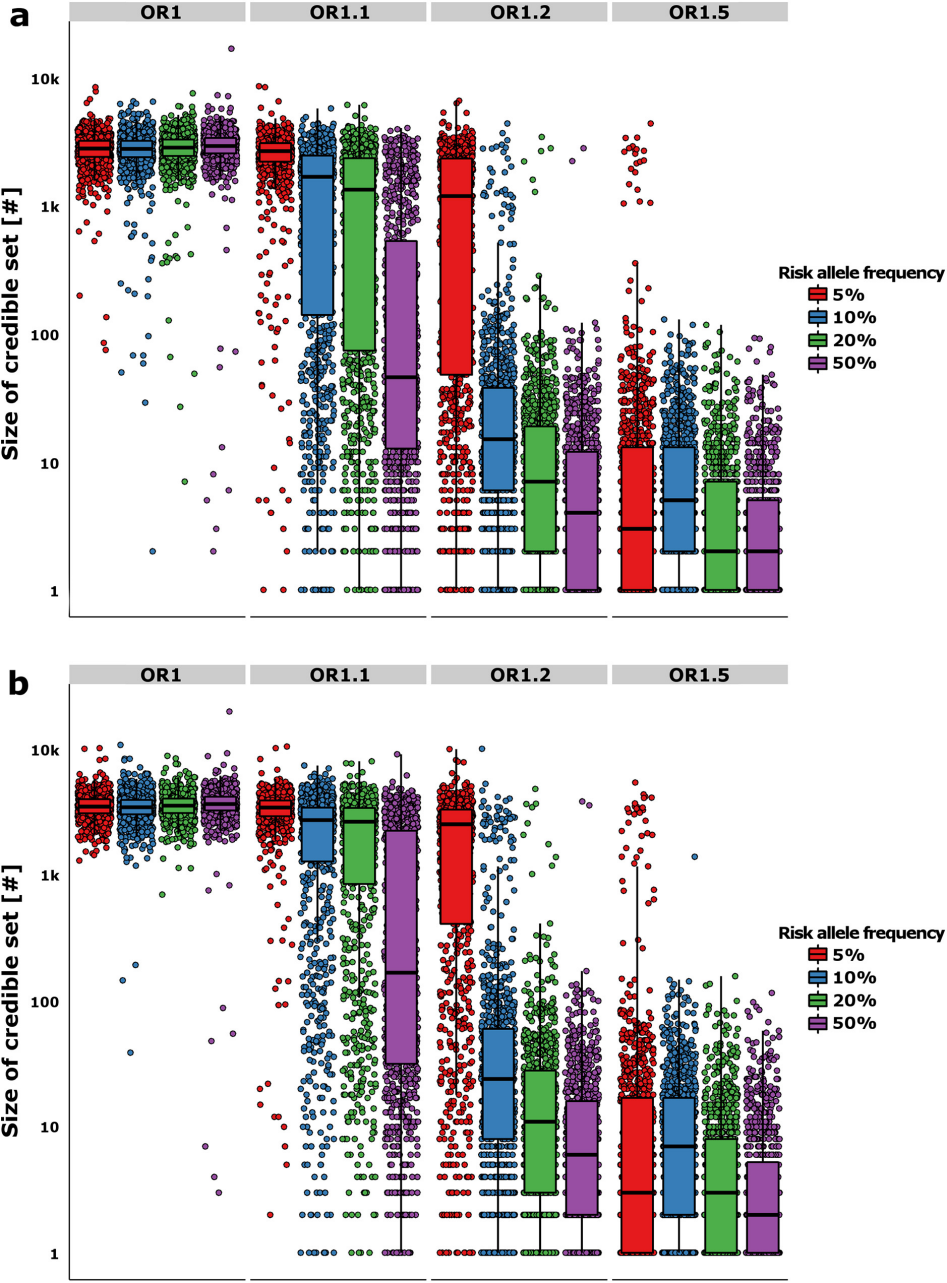
Refinement of the credible sets. The y-axis shows the number of variants in the 95% (a) and 99% (b) likely credible sets. The boxplots show the median and interquartile range of the simulations, while each point denotes a single “replicate”. The color of the boxplots/points denotes the RAF of the simulated causal variant, while each panel is split by the effect size along the horizontal plane.

Next, we evaluated the properties of credible sets for fine-mapping over a range of association models. When the association signal was strong—e.g. RAF 50% and OR 1.5—the median 95% credible set contained only a single variant (and two in the median 99% set). Ninety percent of all replicates generated credible sets containing <11 (95%) and <14 variants (99%) (Fig 2A and 2B). In the simulations with lesser power to detect association (RAF 10%, OR 1.2–58.5% power), the median set contained 15 variants at 95% and 24 variants at the 99% posterior probability cut-off, but the range was far larger (the 90^th^ centiles now were 96 and 183 variants–Fig 2A and 2B). Overall, the size of the credible set had a strong log-negative correlation with the power to detect genome-wide association (*r* ^*2*^ = 0.61 and *r* ^*2*^ = 0.68 for the 95% and 99% credible sets–S2 Fig); at lower power, fine-mapping ability was mostly attenuated.

We then asked how many of the loci could be successfully fine-mapped. For this purpose, we deemed resolution of the credible set to fewer than ten variants to constitute success, on the grounds that this is a tractable number that would encourage researchers to undertake detailed functional evaluation. The success-rate for the 95% credible sets ranged from 88.4% for RAF 50% variants (OR = 1.5) to 36.9% for RAF 10% (OR = 1.2) variants in simulations with >50% power. The equivalent figures for the 99% credible sets were 85.5% and 28.4% respectively. In the least well performing simulation setting overall (RAF 5% at OR = 1.1), 95.3% of the replicates did not fine-map at all, with the credible sets containing > 500 variants within the 1Mb region. However, the results showed that the approximate Bayesian fine-mapping approach provided both good coverage of the causal variant and was often able to successfully refine loci to credible sets of a size that would support exhaustive functional follow-up.

### Impact of different fine-mapping strategies on refinement results

**Fig 3:**
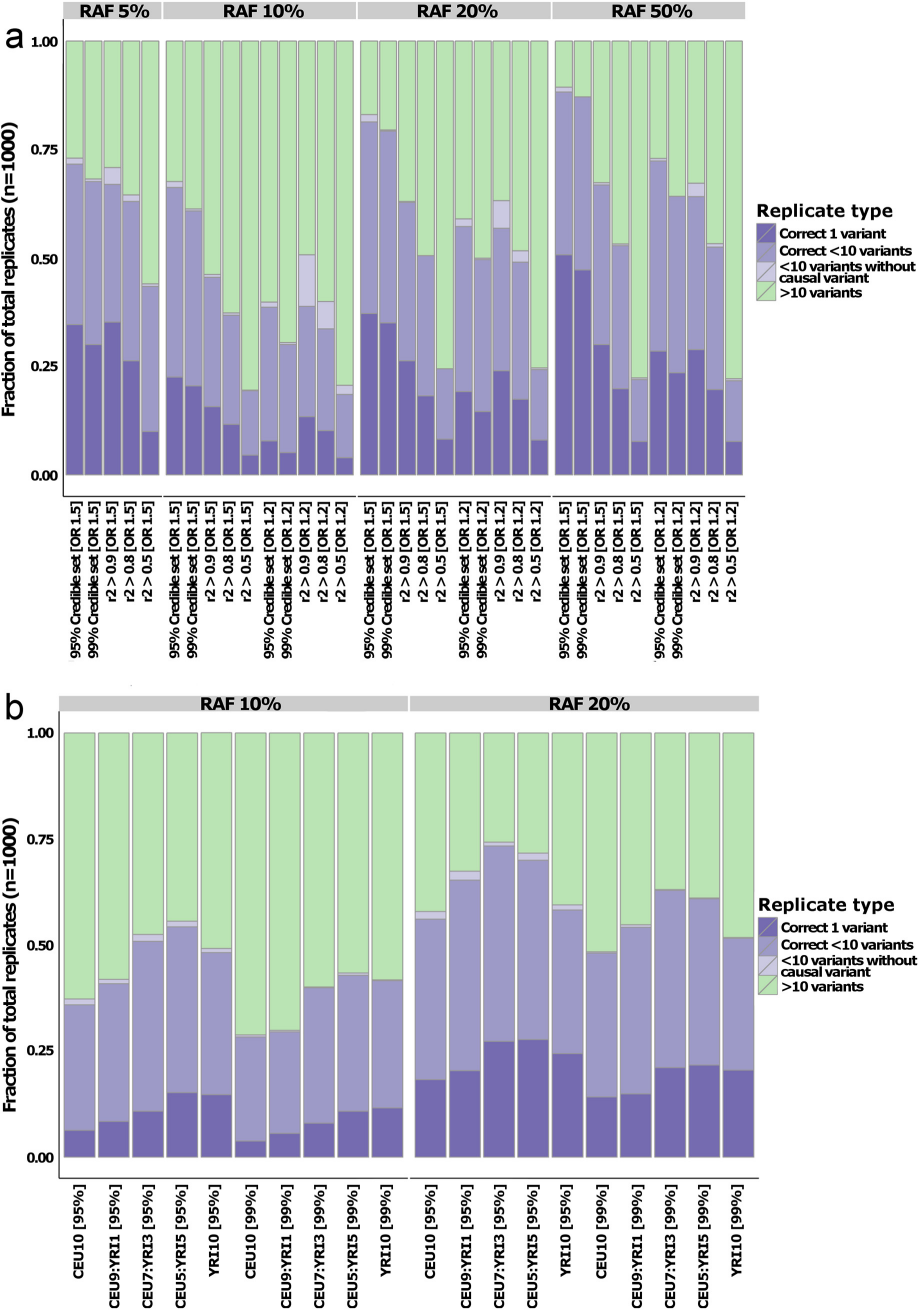
Quantifying fine-mapping success. Fraction of the simulations where the fine-mapped set is reduced to fewer than 10 variants for the comparison with *r2*-derived sets (a) and multi-ethnic study designs (b). Colors denote whether the set contains the causal variants and only one variant (dark purple), causal variant and fewer than 10 variants (medium purple), fewer than 10 variants but not the causal variant (light purple), or more than 10 variants (green). In both panels, the data is split by risk allele frequency (RAF) on the horizontal plane. Panel (a) is grouped by odds ratio (OR) on the x-axis, whereas the OR was set to 1.2 in all the multi-ethnic simulations.

To establish the impact of different fine-mapping strategies and causal variant scenarios, we compared the approximate Bayesian fine-mapping approach to a more simplistic strategy of retaining all variants with *r* ^*2*^ to the lead variant exceeding some pre-defined threshold (0.5, 0.8 or 0.9). We found that, in simulations with great power to detect genome-wide significant association, the approximate Bayesian approach resulted in smaller credible sets than sets based on *r* ^*2*^ thresholds (e.g. 0.8-fold reduction of *r* ^*2*^ >0.9 variant set containing fewer than 10 variants compared to 95% credible sets at RAF 50% and OR 1.5 –Fig 3A). At replicates with less strong association signal, stringent *r* ^*2*^ thresholds (0.9, and, to a lesser extent, 0.8) often resulted in smaller fine-mapped variant sets than those generated by the approximate Bayesian approach (Fig 3A). However, the smaller sets for the *r* ^*2*^ approach at lower OR and RAF were accompanied by a substantial increase in the false-positive rate (e.g. 24% of *r* ^*2*^ >0.9 sets with fewer than 10 variants do not actually contain the causal variant at RAF 10% and OR 1.2 –Fig 3A). This increase in false-positives was not seen using approximate Bayesian credible sets (Fig 3A).

### Trans-ethnic fine-mapping

Trans-ethnic datasets are widely promoted as a useful adjunct to fine-mapping approaches. We therefore explored the value of adding samples from a distinct ancestral group, focusing on individuals of African descent. The reduced LD typically observed in African-descent populations should support improved fine-mapping, at least at those loci where the same causal variants are segregating across ancestries. To investigate this, we took the same design of 10 studies, each of 1000 cases and 1000 controls, but generated some to all of these studies (1, 3, 5 or 10) using the Yoruba (YRI) component of 1000 Genomes. The simulated causal variant was kept identical to that in the CEU simulations, and with the same effect size. However, the RAF for the causal variant in the YRI haplotypes diverged, and was typically lower than that observed in CEU. We focused these simulations on the less extreme GWAS scenarios–causal variant RAFs of 10%, 20% at OR = 1.2 (defined by CEU data)–and saw a marked improvement in fine-mapping resolution compared to the equivalent CEU-only study. For example, in an analysis of 5 CEU and 5 YRI samples, the probability that the lead GWAS variant was also the causal variant increased 1.7-fold (27% to 46%; S3 Fig) for RAF of 10% and 1.3-fold (45% to 60%; S3 Fig) for RAF of 20%. The increased fine-mapping resolution was also reflected in the credible sets: the number of successfully refined loci (< 10 variants) increased on average by 1.5-fold (RAF 10%) and 1.3-fold (RAF 20%) in the 50% YRI design compared to a CEU-only study (Fig 3B). Failure to see incremental improvements in fine-mapping resolution as YRI proportion increased (the results for 50% YRI are better than for 100% YRI) could reflect the advantage in signal localization possible from analyses that can benefit from the divergent LD patterns across the two ethnicities. However, it might also be due to the causal variant RAF being derived from the CEU population: as demonstrated in the broad evaluation of the properties of approximate Bayesian fine-mapping above, ABFs are dependent on power, and substantially lower risk allele frequencies in the YRI population result in loss of association signal. Overall, these results confirm the utility of multi-ethnic design for fine-mapping purposes, at least when the assumption of a shared causal allele is realized.

### Inclusion of functional priors in fine-mapping

With the emergence of genome-wide functional annotations, such as those generated by the ENCODE, GENCODE and NIH Roadmap Epigenomics projects, these data can now be used to inform fine-mapping [14]. We considered whether including such prior information in the approximate Bayesian framework would further improve its performance. For illustration purposes, we chose to set an elevated prior on exonic variants, but any other functional annotation for which there is evidence of disproportionate functional impact, such as islet enhancers in type 2 diabetes [15], could also have been used. We simulated one thousand 1Mb random regions containing a causal coding variant of OR 1.2 and RAF 10% as described (see Methods), but in these replicates the causal variant was limited to exonic sequence. This was followed by fine-mapping with or without a functional prior of ten-fold greater probability on coding variants–roughly corresponding to the fold over-representation of coding variants observed in GWAS studies [16]. The weighted approach resulted in a 1.4-fold reduction in the 95%, and 1.5-fold reduction in the 99% credible set size compared to non-weighted sets derived from the same data (S4A Fig). In line with this, there was an increase in the percentage of successfully fine-mapped loci in the weighted scenario, with 35.8% of the weighted 95% credible sets containing fewer than ten variants, compared to 26.9% for the non-weighted sets (S4B Fig). For the 99% credible sets, the rates of successful fine-mapping were 25.4% and 17.0% respectively (S4B Fig). Using a model-based approach, it has been shown that, when causal variation was limited to small genomic regions (100 loci of 10kb each) containing a large amount of the total trait variance (25%)[16], functional priors increased fine-mapping performance. Our results show that similar improvements in fine-mapping results can be obtained using the approximate Bayesian approach at loci containing variants which explain much less of the total heritability.

### Fine-mapping in loci with multiple causal variants

All the above simulations assume a single causal variant at each locus. To determine how the approximate Bayesian approach would deal with the presence of multiple signals at a locus, unbeknown to the investigator, we simulated 1000 random regions containing two distinct causal variants of equal RAF (10%) and effect size (OR 1.2). As might be expected, the size of the 95% credible sets showed a 1.3-fold increase (median 15 versus 19 variants with a single versus two causal variants present), whereas the 99% credible sets increased by 1.5-fold (24 versus 35 variants–S3C Fig). This was accompanied by a reduction in the number of successfully refined loci (1.3-fold; S3D Fig). However, this still meant that at 30.5% of replicate loci at least one causal variant was included in a 95% credible set containing fewer than ten variants–only in 5.2% were both causal variants present in such a set. In practice, a preliminary round of conditional analysis would allow such instances of multiple association signals at the same locus to be detected, and fine-mapping efficiency could then be maximized by considering each of the component signals separately.

### Fine-mapping behavior on empirical data

Finally, we used an empirical data set to investigate if we could recapitulate the effectiveness of the fine-mapping observed in the simulations. We applied the approximate Bayesian approach (as described in the Methods) to data for twenty six non-MHC loci genotyped at high density (using the Illumina Immunochip with subsequent 1000 Genomes European ancestry imputation) for 9,049 cases and 13,607 controls of European origin from a published association study for ankylosing spondylitis (AS) [5]. The mean RAF 32% (range 5–48%) and OR 1.17 (1.11–1.65) of the reported lead variants at these loci were broadly in line with the simulated datasets. Approximate Bayesian fine-mapping resulted in median 95% and 99% credible sets containing 20 (1–295) and 38 (2–1,113) variants, respectively (S1 Table).

**Fig 4:**
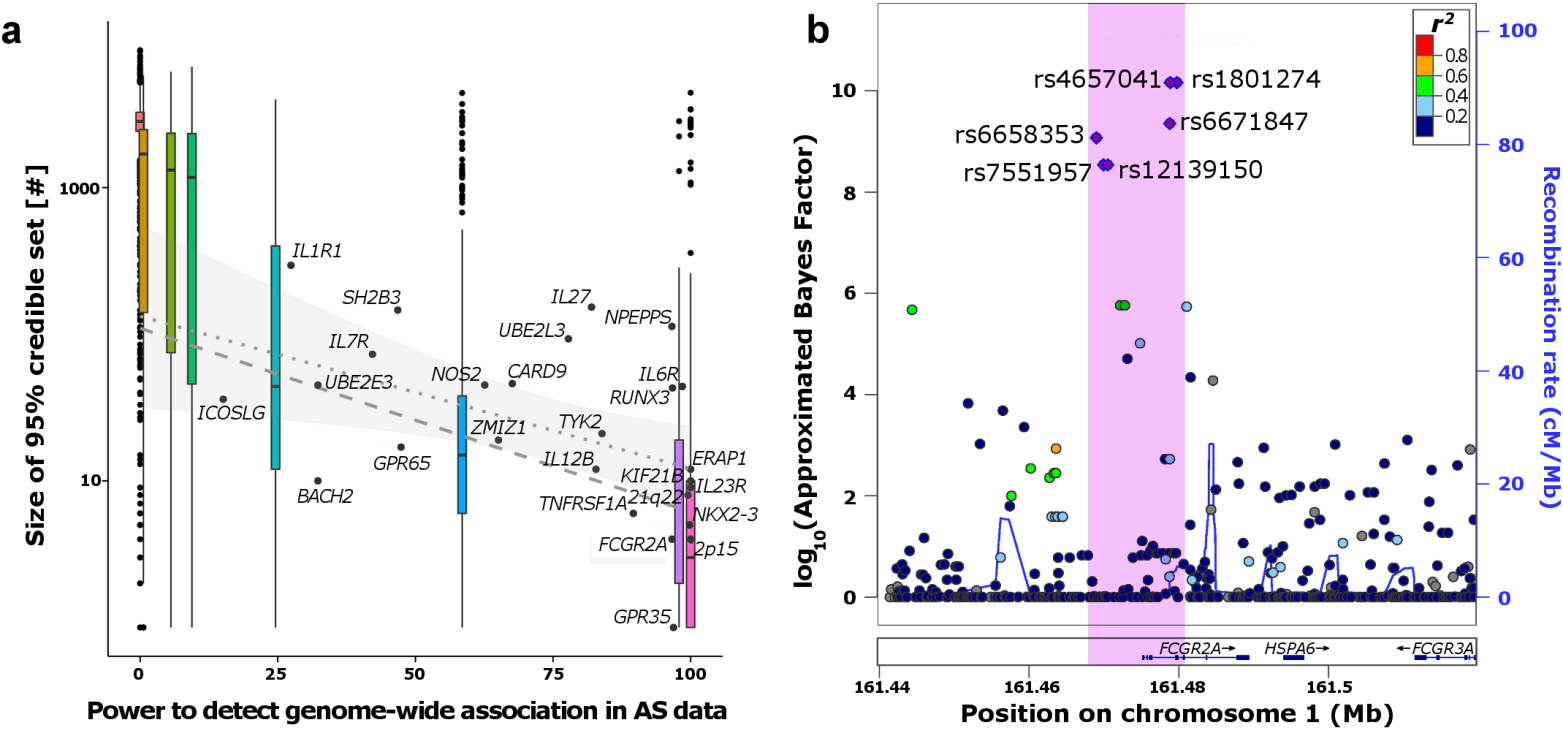
Fine-mapping the ankylosing spondylitis (AS) regions. a) Correlation between predicted power to detect genome-wide association signals and size of the 95% credible sets. Boxplots represent the distribution of the simulations at the respective power of each RAF/OR setting. The labelled dots show the distribution of the empirical AS data. Regression lines in the range of predicted power of the AS loci (15–99.9%) are derived from the simulations (dashed) and AS loci (dotted with confidence interval of regression line). b) Example *FCGR2A* region where the 99% credible set (purple dots) could be fine-mapped to a small region (pink) containing few variant.

In line with the simulations, the success of fine-mapping across these 26 loci was correlated with the effect size and RAF at each locus (and hence with the power to detect the locus in association testing–Fig 4A). Several of the loci that lie most outside the interquartile range of observed 95% credible set sizes in the simulations show extensive LD: index variants at the *IL27*, *NPEPPS* and *SH2B3* loci all are in LD (*r* ^*2*^>0.1) with a proportion of variants in the 1Mb interval that compare to the top 10 quantile of simulated loci.

Seven of the twenty six loci (27%) were reduced to fewer than ten 95% credible variants (S1 Table). Overall, twenty four of the twenty six credible sets derived from our European-only analysis included the lead variant reported in the larger multi-ethnic meta-analysis from Cortes *et al*. [5]. One example of our approximate Bayesian approach revealing strong functional candidates can be found at the *FCGR2A* locus. The 99% credible set for this locus, which is associated not only with AS, but with a range of other autoimmune conditions [5,17], contained only six variants spanning 11kb of the promoter region and first 3 exons of *FCGR2A* (Figs 4B and S5). One of these six variants within the credible set was a missense variant (rs1801274) in *FCGR2A*, which encodes an immunoglobulin Fc receptor gene found on the surface of many immune response cells.

In conclusion, simulated and empirical data analyses demonstrate that fine-mapping represents an effective strategy for causal variant localization, at a subset of loci at least. The simulations also emphasize that trans-ethnic study designs can improve resolution further. It is still true that the vast majority of GWAS data is generated from individuals of European descent, and even trans-ethnic studies such as Cortes *et al*. generally contains only a modest non-European contribution (~14% of the total sample size). Therefore, empirical assessment of the full value of trans-ethnic over ethnic-specific fine-mapping will ultimately depend on the generation of large scale non-European GWAS data.

The simulations clearly show that fine-mapping is sample size dependent, so the utility of this approach is dictated by the availability of large-scale data. Fine-mapping resolution is most precise at strong association signals, such that for a variant with allelic OR of 1.2 and RAF of 50%, fine-mapping in 10,000 cases and 10,000 controls of European origin can be expected to reduce the credible set to fewer than ten variants at around 60% of loci. For less strong signals, resolution is typically less good, but, even here, the generation of well-calibrated credible sets facilitates integration with genomic annotation data. Indeed, one advantage of the approximate Bayesian framework used here is that prior information from external data resources such as ENCODE [18], can readily be included to upweight highly-annotated variants. This can help prioritize specific signals and aid the selection of appropriate functional assays for follow-up. In addition, fine-mapping strategies utilizing ABFs are computationally inexpensive and can be applied to publicly available association summary statistics from GWAS data without the need to access individual level genotypes. This should allow rapid deployment of fine-mapping analyses to existing GWAS data sets, and encourage efforts to convert common variant GWAS data into an improved understanding of disease biology.

## Methods

### Design of the simulations pipeline

We used sequence data from 85 CEU individuals from 1000 Genomes Phase 1, release 3[4] (single nucleotide variants only), to simulate 1Mb autosomal genomic regions with HAPGEN2 [19]–an algorithm which resamples known haplotypes and thus maintains experimentally-derived LD patterns. When evaluating the effect of multi-ethnic study design on the fine-mapping resolution, one, three, five or all of the ten studies were simulated based on haplotypes from 88 YRI rather than 85 CEU individuals from 1000 Genomes Phase 1, release 3.

One thousand non-overlapping 1Mb regions were randomly selected from the mappable human genome reference. In the center of each region, causality was assigned to a single causal variant of specified risk allele frequency (RAF; 50%, 20%, 10% or 5%) with an additive phenotypic effect (OR 1.5, 1.2, 1.1 and 1.0, the last corresponding to the null model of no association). In the evaluation of the impact on fine-mapping of multiple causal variants in a region, 1000 regions containing two distinct variants (CEU *r* ^*2*^<0.05) with a MAF~10% in the central 750kb of the 1Mb interval were selected. Haplotypes were simulated using HAPGEN2 with, with each of the two variants assigned an additive phenotypic effect (OR = 1.2). For each RAF/OR parameter setting, genotypes were generated for ten “studies” of 1,000 cases and 1,000 controls for each of the one thousand “replicate” genomic regions. Case-control association analysis was performed by logistic regression under an additive model, implemented in SNPTEST, and summary statistics from the ten studies aggregated by fixed-effects meta-analysis using GWAMA [20].

We performed these analyses using an unfiltered set of 1000 Genome-derived genotypes, equivalent to an association study performed on genome-wide sequence data (“gold standard” scenario). To represent a more typical fine-mapping scenario, variants from each study were downsampled to the content of an Illumina HumanOmni2.5 BeadChip array (“GWAS” scenario). This is also a similar density to that achieved within established GWAS regions using recent custom arrays such as Metabochip [13] or Immunochip [21]. To capture the effects of genotyping failure, downsampling was performed with and without 5% random variant failure per cohort (n = 1000 cases or controls) in each simulated GWAS (“GWAS with failure” scenario). These GWAS genotypes were then imputed (using IMPUTE2 [22]) up to the 1000G Phase 1, all ancestries release 3 panel before analysis as above. Only well-imputed variants (INFO score >0.4) with a MAF > = 1% were included in further analyses.

### Deriving approximate Bayesian factors from GWAS summary statistics

While privacy concerns mean that individual-level genotype data for GWAS are generally unavailable to researchers, large amounts of summary statistics are available online. Summary data do not allow full specification of disease and null models, but the information contained within can be used in a Bayesian framework by instead approximating Bayes’ factors as proposed by Wakefield in 2007 [12]. This approximation assumes that the likelihood distribution for association is summarized by the regression parameters with a prior for association centered on 0 (which corresponds to the null of no association) and variance dependent on *W*, which describes the strength of association conditional on its existence. The value for *W* is set at 0.4, which equates to a 95% belief that the relative risk corresponding to departure from the null model is less than 1.5.

As demonstrated by Wakefield [12], this results in an equation for the approximate Bayes Factor (ABF) given as

$$ABF=\frac{1}{\sqrt{1-r}}\exp\left(-\frac{Z^{2}}{2}r\right)$$

where *Z* is the *Z*-statistic describing the strength of association derived from the regression and *r* a shrinkage factor

$$r=\frac{W}{V+W}$$

defining the ratio of prior variance to total variance. The resulting ABF is dependent the effect size through *Z* and the power of the study through the variance of the effect *V*. ABFs for variants with missing genotype data were corrected by rescaling variance to the maximum observed effective sample size (provided sample size was within 30% of the maximum observed sample size). Posterior probabilities for association were calculated based on the ABFs all variants in each of the simulated regions.

### Power calculations

For assessing the relation between fine-mapping performance and power to detect genome-wide association, we used Quanto v1.2.4 (available from http://biostats.usc.edu/Quanto.html). The software was run in “Gene only” setting, assuming a disease prevalence of 0.55% in line with that of ankylosing spondylitis. The power for each combination of OR and RAF, for both simulations and empirical data, was calculated individually.

## Supporting Information

S1 Fig Coverage of the causal variant in the credible sets. Percentage of simulations with the causal variant included in the credible set (y-axis) by RAF of the causal variant (x-axis). The figure is split according the OR (horizontal) and simulation scenario (vertical). Colors denote probability cut-off used for the credible sets.
(TIFF)

S2 Fig Correlation between fine-mapping performance and power to detect genome-wide significant association. Predicted power to detect genome-wide association for a disease with prevalence of 0.55% in each of the simulated scenarios (x-axis) versus the size of the 95% (a) and 99% (b) credible sets. The boxplots represent the median and 1^st^ to 3^rd^ interquartile range for all simulations at the given power. The result of the linear regression for y ~ x is shown as the dashed grey line.
(TIFF)

S3 Fig Ranking of the causal variant across simulated trans-ethnic loci. Cumulative percentage of simulations (y-axis) with decreasing ranking of the causal variant amongst all variants in the regions (x-axis) based with a causal variant RAF of 10% (a) and 20% (b). The panels are split along the horizontal by simulation scenario.
(TIFF)

S4 Fig Fine-mapping success with functional priors (a-b) and multiple causal variants (c-d). Left panels show the difference in credible set sizes for the weighted versus unweighted (a) and multiple causal variant (c) scenarios. Boxplots show the median and interquartile range of the simulations, while each point denotes a single replicate. For each category, the data is split by credible set type on the x-axis. The right hand panels show the fraction of the simulations where the fine-mapped set is reduced to fewer than 10 variants in the same order as before. Colors denote whether the credible set contains only one variant (dark purple), causal variant and fewer than 10 variants (medium purple), fewer than 10 variants but not the causal variant (light purple), or more than 10 variants (green).
(TIFF)

S5 Fig Fine-mapping the ankylosing spondylitis *FCGR2A* locus. Zoomplot of the *FCGR2A* locus with–log_10_(*p*-values) rather than log_10_(ABFs) plotted for each variant on the y-axis.
(TIFF)

S1 Note Supplementary Note. Memberships of the International Genetics of Ankylosing Spondylitis (IGAS) Consortium.
(PDF)

S1 Table Fine-mapping results for the twenty six AS loci from Cortes et al. using European-only data. (PDF)

## References

[1] Dickson SP, Wang K, Krantz I, Hakonarson H, Goldstein DB (2010) Rare variants create synthetic genome-wide associations. PLoS Biol 8: e1000294 10.1371/journal.pbio.1000294 20126254 PMC2811148

[2] Hunt KA, Mistry V, Bockett NA, Ahmad T, Ban M, et al (2013) Negligible impact of rare autoimmune-locus coding-region variants on missing heritability. Nature 498: 232–235. 10.1038/nature12170 23698362 PMC3736321

[3] Morris AP, Voight BF, Teslovich TM, Ferreira T, Segre AV, et al (2012) Large-scale association analysis provides insights into the genetic architecture and pathophysiology of type 2 diabetes. Nat Genet 44: 981–990. 10.1038/ng.2383 22885922 PMC3442244

[4] Abecasis GR, Auton A, Brooks LD, DePristo MA, Durbin RM, et al (2012) An integrated map of genetic variation from 1,092 human genomes. Nature 491: 56–65. 10.1038/nature11632 23128226 PMC3498066

[5] Cortes A, Hadler J, Pointon JP, Robinson PC, Karaderi T, et al (2013) Identification of multiple risk variants for ankylosing spondylitis through high-density genotyping of immune-related loci. Nat Genet 45: 730–738. 10.1038/ng.2667 23749187 PMC3757343

[6] Maller JB, McVean G, Byrnes J, Vukcevic D, Palin K, et al (2012) Bayesian refinement of association signals for 14 loci in 3 common diseases. Nat Genet 44: 1294–1301. 10.1038/ng.2435 23104008 PMC3791416

[7] Beecham AH, Patsopoulos NA, Xifara DK, Davis MF, Kemppinen A, et al (2013) Analysis of immune-related loci identifies 48 new susceptibility variants for multiple sclerosis. Nat Genet. 10.1038/ng.2770 PMC3832895 24076602

[8] Rivas MA, Beaudoin M, Gardet A, Stevens C, Sharma Y, et al (2011) Deep resequencing of GWAS loci identifies independent rare variants associated with inflammatory bowel disease. Nat Genet 43: 1066–1073. 10.1038/ng.952 21983784 PMC3378381

[9] Wu Y, Waite LL, Jackson AU, Sheu WH, Buyske S, et al (2013) Trans-ethnic fine-mapping of lipid loci identifies population-specific signals and allelic heterogeneity that increases the trait variance explained. PLoS Genet 9: e1003379 10.1371/journal.pgen.1003379 23555291 PMC3605054

[10] Mahajan A, Go MJ, Zhang W, Below JE, Gaulton KJ, et al (2014) Genome-wide trans-ancestry meta-analysis provides insight into the genetic architecture of type 2 diabetes susceptibility. Nat Genet 46: 234–244. 10.1038/ng.2897 24509480 PMC3969612

[11] Gong J, Schumacher F, Lim U, Hindorff LA, Haessler J, et al (2013) Fine Mapping and Identification of BMI Loci in African Americans. Am J Hum Genet 93: 661–671. 10.1016/j.ajhg.2013.08.012 24094743 PMC3791273

[12] Wakefield J (2007) A Bayesian measure of the probability of false discovery in genetic epidemiology studies. Am J Hum Genet 81: 208–227. 17668372 10.1086/519024 PMC1950810

[13] Voight BF, Kang HM, Ding J, Palmer CD, Sidore C, et al (2012) The metabochip, a custom genotyping array for genetic studies of metabolic, cardiovascular, and anthropometric traits. PLoS Genet 8: e1002793 10.1371/journal.pgen.1002793 22876189 PMC3410907

[14] Trynka G, Sandor C, Han B, Xu H, Stranger BE, et al (2013) Chromatin marks identify critical cell types for fine mapping complex trait variants. Nat Genet 45: 124–130. 10.1038/ng.2504 23263488 PMC3826950

[15] Pasquali L, Gaulton KJ, Rodriguez-Segui SA, Mularoni L, Miguel-Escalada I, et al (2014) Pancreatic islet enhancer clusters enriched in type 2 diabetes risk-associated variants. Nat Genet 46: 136–143. 10.1038/ng.2870 24413736 PMC3935450

[16] Kichaev G, Yang WY, Lindstrom S, Hormozdiari F, Eskin E, et al (2014) Integrating functional data to prioritize causal variants in statistical fine-mapping studies. PLoS Genet 10: e1004722 10.1371/journal.pgen.1004722 25357204 PMC4214605

[17] Jostins L, Ripke S, Weersma RK, Duerr RH, McGovern DP, et al (2012) Host-microbe interactions have shaped the genetic architecture of inflammatory bowel disease. Nature 491: 119–124. 10.1038/nature11582 23128233 PMC3491803

[18] Dunham I, Kundaje A, Aldred SF, Collins PJ, Davis CA, et al (2012) An integrated encyclopedia of DNA elements in the human genome. Nature 489: 57–74. 10.1038/nature11247 22955616 PMC3439153

[19] Su Z, Marchini J, Donnelly P (2011) HAPGEN2: simulation of multiple disease SNPs. Bioinformatics 27: 2304–2305. 10.1093/bioinformatics/btr341 21653516 PMC3150040

[20] Magi R, Morris AP (2010) GWAMA: software for genome-wide association meta-analysis. BMC Bioinformatics 11: 288 10.1186/1471-2105-11-288 20509871 PMC2893603

[21] Cortes A, Brown MA (2011) Promise and pitfalls of the Immunochip. Arthritis Res Ther 13: 101 10.1186/ar3204 21345260 PMC3157635

[22] Howie BN, Donnelly P, Marchini J (2009) A flexible and accurate genotype imputation method for the next generation of genome-wide association studies. PLoS Genet 5: e1000529 10.1371/journal.pgen.1000529 19543373 PMC2689936

