# Supplementary figures and images for: Evaluating the Performance of Fine-Mapping Strategies at Common Variant GWAS Loci
Source: PLoS Genet. 2015 Sep 25;11(9):e1005535. doi: 10.1371/journal.pgen.1005535 (PMC4583479; doi:10.1371/journal.pgen.1005535)

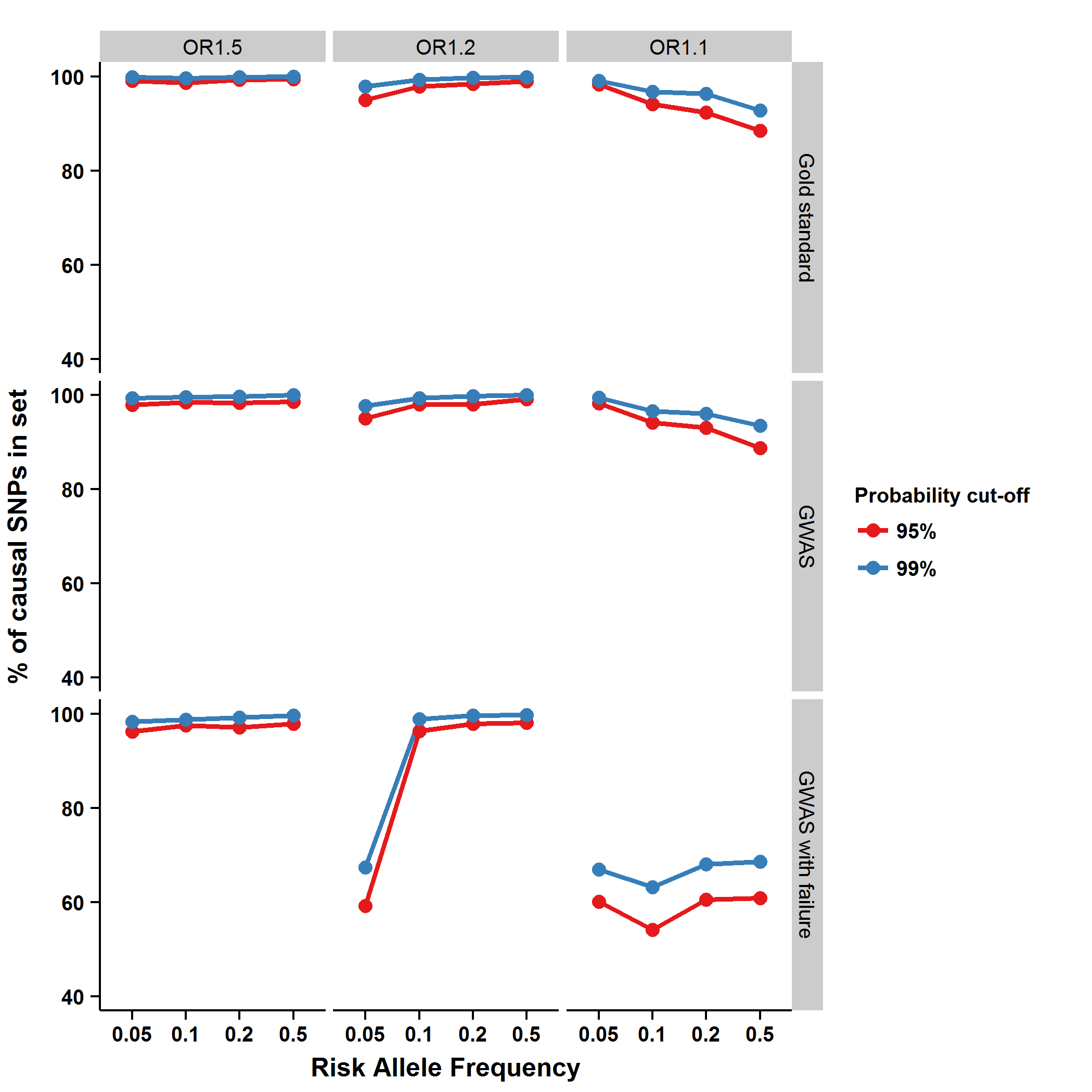

Supplement: S1 Fig — Percentage of simulations with the causal variant included in the credible set (y-axis) by RAF of the causal variant (x-axis). The figure is split according the OR (horizontal) and simulation scenario (vertical). Colors denote probability cut-off used for the credible sets. (TIFF) [file pgen.1005535.s001.tiff]

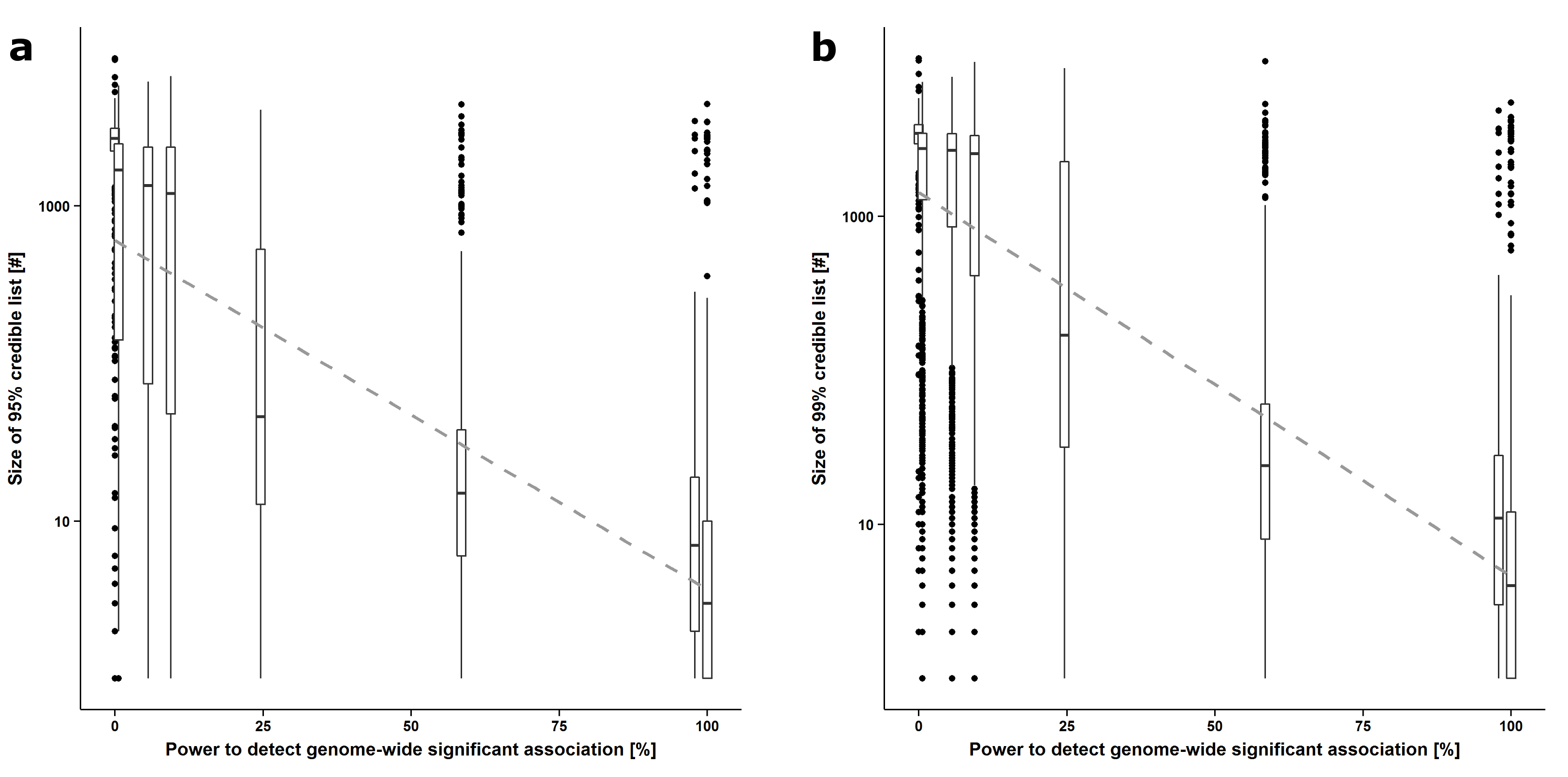

Supplement: S2 Fig — Predicted power to detect genome-wide association for a disease with prevalence of 0.55% in each of the simulated scenarios (x-axis) versus the size of the 95% (a) and 99% (b) credible sets. The boxplots represent the median and 1st to 3rd interquartile range for all simulations at the given power. The result of the linear regression for y ~ x is shown as the dashed grey line. (TIFF) [file pgen.1005535.s002.tiff]

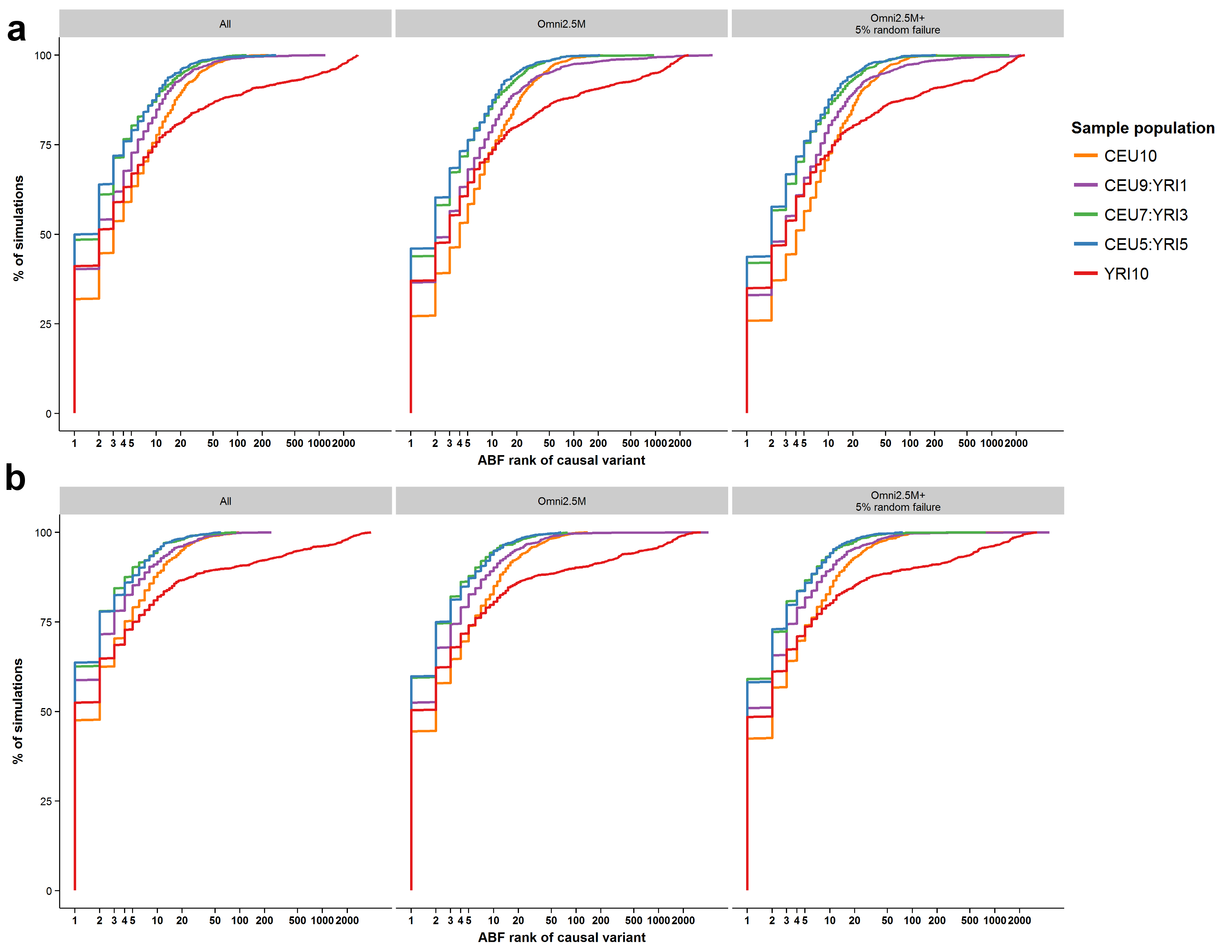

Supplement: S3 Fig — Cumulative percentage of simulations (y-axis) with decreasing ranking of the causal variant amongst all variants in the regions (x-axis) based with a causal variant RAF of 10% (a) and 20% (b). The panels are split along the horizontal by simulation scenario. (TIFF) [file pgen.1005535.s003.tiff]

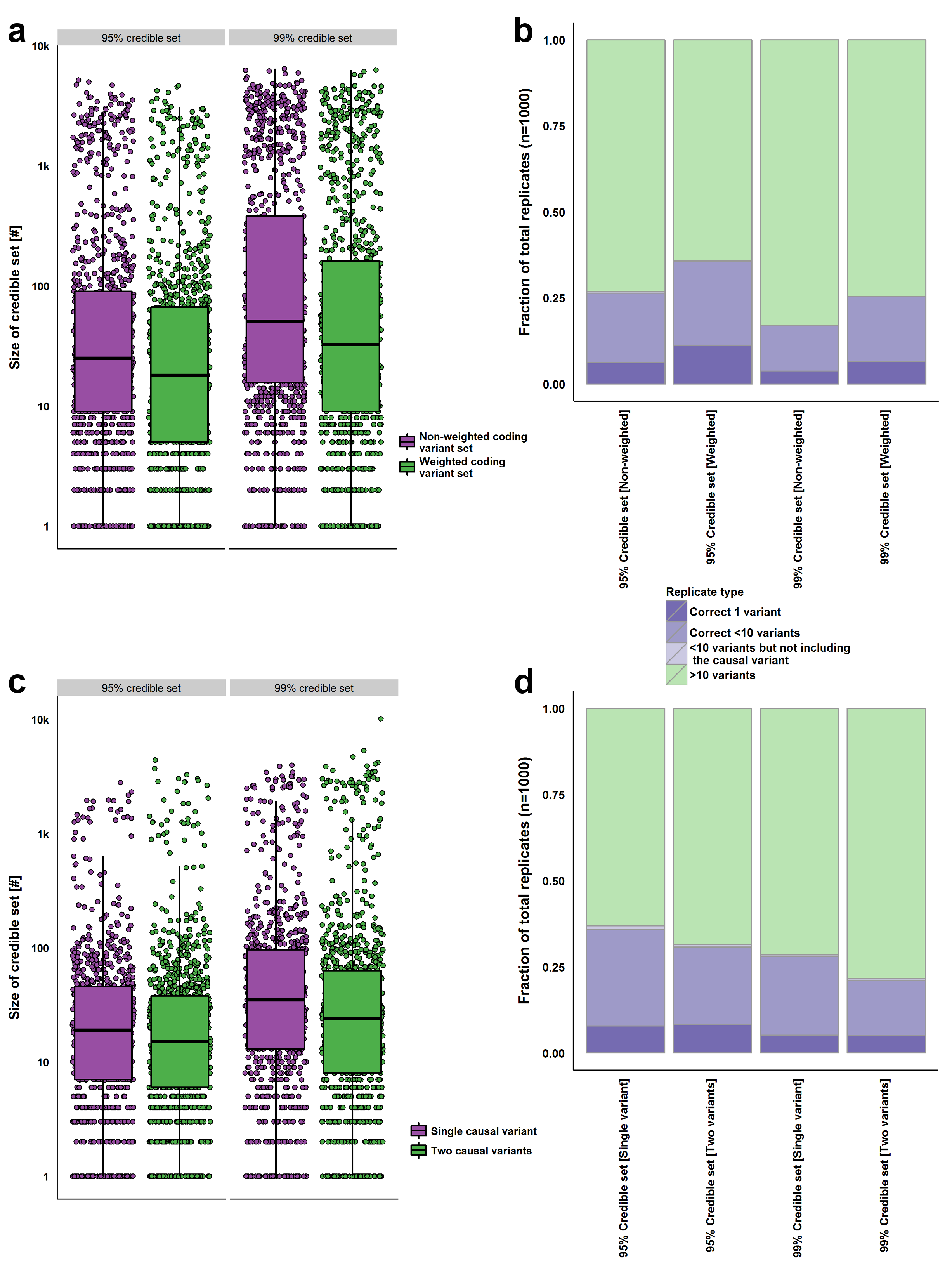

Supplement: S4 Fig — Left panels show the difference in credible set sizes for the weighted versus unweighted (a) and multiple causal variant (c) scenarios. Boxplots show the median and interquartile range of the simulations, while each point denotes a single replicate. For each category, the data is split by credible set type on the x-axis. The right hand panels show the fraction of the simulations where the fine-mapped set is reduced to fewer than 10 variants in the same order as before. Colors denote whether the credible set contains only one variant (dark purple), causal variant and fewer than 10 variants (medium purple), fewer than 10 variants but not the causal variant (light purple), or more than 10 variants (green). (TIFF) [file pgen.1005535.s004.tiff]
