# Supplementary material for: Evaluating the Performance of Fine-Mapping Strategies at Common Variant GWAS Loci
Source: PLoS Genet. 2015 Sep 25;11(9):e1005535. doi: 10.1371/journal.pgen.1005535 (PMC4583479; doi:10.1371/journal.pgen.1005535)
Supplement: S1 Note — Memberships of the International Genetics of Ankylosing Spondylitis (IGAS) Consortium. (PDF) [file pgen.1005535.s006.pdf]

Supplementary Note

Memberships of the International Genetics of Ankylosing Spondylitis (IGAS) Consortium

The IGAS Consortium consists of the following people (excluding A.C. and M.A.B. already listed as authors):

Johanna Hadler[1], Jenny P Pointon[2], Philip C Robinson[1], Tugce Karaderi[2], Paul Leo[1], Katie Cremin[1], Karena Pryce[1], Jessica Harris[1], Seunghun Lee[3], Kyung Bin Joo[3], Seung-Cheol Shim[4], Michael Weisman[5], Michael Ward[6], Xiaodong Zhou[7], Henri-Jean Garchon[8, 9], Gilles Chiocchia[8], Johannes Nossent[10, 11], Benedicte A Lie[12, 13], Øystein Førre[14], Jaakko Tuomilehto[15], Kari Laiho[16], Lei Jiang[17], Yu Liu[17], Xin Wu[17], Linda A Bradbury[1], Dirk Elewaut[18], Ruben Burgos-Vargas[19], Simon Stebbings[20], Louise Appleton[2], Claire Farrah[2], Jonathan Lau[2], Tony J Kenna[1], Nigil Haroon[21], Manuel A Ferreira[22], Jian Yang[1], Juan Mulero[23], Jose Luis Fernandez-Sueiro[24], Miguel A Gonzalez-Gay[25], Carlos Lopez-Larrea[26], Panos Deloukas[27], Peter Donnelly[28], Australo-Anglo-American Spondyloarthritis Consortium (TASC), Groupe Française d'Etude Génétique des Spondylarthrites (GFEGS), Nord-Trøndelag health study (HUNT), Spondyloarthritis Research Consortium of Canada (SPARCC), Wellcome Trust Case-Control Consortium 2 (WTCCC2), Lianne Gensler[29], Paul Bowness[2], Karl Gafney[30], Hill Gaston[31], Dafna D Gladman[32, 33, 34], Proton Rahman[35], Walter P Maksymowych[36], Huji Xu[17], J Bart A Crusius[37], Irene E van der Horst-Bruinsma[38], Chung-Tei Chou[39, 40], Raphael Valle-Oñate[41], Consuelo Romero-Sánchez[41], Inger Myrnes Hansen[42], Fernando M Pimentel-Santos[43], Robert D Inman[21], Vibeke Videm[44, 45], Javier Martin[46], Maxime Breban[8, 9], John D Reveille[7], David M Evans [47], Tae-Hwan Kim[3], and Bryan Paul Wordsworth[2]

[1]University of Queensland Diamantina Institute, Translational Research Institute, Brisbane, Australia
[2]National Institute for Health Research (NIHR) Oxford Musculoskeletal Biomedical Research Unit, Nuffield Orthopaedic Centre, Headington, Oxford, United Kingdom
[3]Department of Rheumatology, Hanyang University Hospital for Rheumatic Diseases, Seoul, Republic of Korea
[4]Division of Rheumatology, Department of Medicine, Eulji University Hospital, Daejeon, Republic of Korea
[5]Department of Medicine/Rheumatology, Cedars-Sinai Medical Center, Los Angeles, California, USA
[6]National Institute of Arthritis and Musculoskeletal and Skin Diseases, National Institutes of Health, Bethesda, Maryland, USA
[7]Rheumatology and Clinical Immunogenetics, University of Texas Health Science Center at Houston, Houston, Texas, USA
[8]Institut Cochin, Université Paris-Descartes, CNRS (UMR 8104), INSERM U1016, France
[9]Ambroise Paré Hospital, Assistance Publique Hôpitaux de Paris, Versailles-Saint-Quentin en Yvelines University, Boulogne-Billancourt, France
[10]University Hospital North Norway, Tromso, Norway
[11]Division of Medicine, Royal Darwin Hospital, Darwin, Australia
[12]Department of Medical Genetics, University of Oslo and Oslo University Hospital, Oslo, Norway
[13]Department of Immunology, Oslo University Hospital, Oslo, Norway
[14]Department of Rheumatology, University Hospital Oslo, Oslo, Norway
[15]Department of Epidemiology and Health Promotion, National Public Health Institute, Helsinki, Finland
[16]Paijat-Hame Central Hospital, Lahti, Finland

[17]Department of Rheumatology and Immunology, Shanghai Changzheng Hospital, Second Military Medical University, Shanghai 200003, China
[18]Department of Rheumatology, Gent University Hospital, Gent, Belgium
[19]Department of Rheumatology, Hospital General de México, Faculty of Medicine, Universidad Nacional Autónoma de México, México, México
[20] Department of Medicine, Dunedin School of Medicine, University of Otago, Dunedin, New Zealand
[21] Division of Rheumatology, Toronto Western Hospital, Toronto, Canada
[22]Queensland Institute of Medical Research, Royal Brisbane Hospital, Herston, Australia
[23]Rheumatology Department, Hospital Puerta de Hierro, Madrid, Spain
[24]Rheumatology Department, Complejo Hospitalario La Coruña, INIBIC, La Coruña, Spain
[25]Rheumatology Department, Hospital Marques de Valcecillas, IFIMAV, Santander, Spain
[26]Department of Immunology, Asturias Central University Hospital, Oviedo, Spain
[27]Wellcome Trust Sanger Institute, Cambridge, United Kingdom
[28]Wellcome Trust Centre for Human Genetics, University of Oxford, Oxford, United Kingdom
[29]University of California, San Francisco, 400 Parnassus Avenue, Box 0326, San Francisco, CA 94143-0326.
[30]Department of Rheumatology, Norfolk and Norwich University Hospital, Norwich, United Kingdom
[31]Department of Medicine, University of Cambridge, Addenbrookes Hospital, Cambridge, United Kingdom
[32]Division of Rheumatology, University of Toronto, Toronto, Canada
[33]Toronto Western Research Institute, Toronto, Canada
[34]Psoriatic Arthritis Program, University Health Network
[35]Memorial University of Newfoundland, Newfoundland, Canada
[36]Department of Medicine, University of Alberta, Canada
[37]Laboratory of Immunogenetics, Department of Medical Microbiology and Infection Control, VU University Medical Center, Amsterdam, The Netherlands
[38]Department of Rheumatology, VU University Medical Centre, Amsterdam, Netherlands
[39]Division of Allergy, Immunology, Rheumatology, Department of Medicine, Taipei Veterans General Hospital, Taipei, Taiwan
[40]School of Medicine, National Yang-Ming University, Taipei, Taiwan
[41]Spondyloarthropaty Group-Division of Rheumatology, Hospital Militar Central/ Universidad de La Sabana, Colombia
[42]Helgelandssykehuset, Mo i Rana, Norway
[43]Chronic Diseases Research Centre (CEDOC), Faculdade de Ciências Médicas, Universidade Nova de Lisboa, Portugal
[44]Department of Laboratory Medicine, Children's and Women's Health, Norwegian University of Science and Technology, Trondheim, Norway
[45]Department of Immunology and Transfusion Medicine, Tronheim University Hospital, Norway
[46]Instituto de Parasitología y Biomedicina López-Neyra, Consejo Superior de Investigaciones Científicas, Granada, Spain
[47]MRC Centre for Causal Analyses in Translational Epidemiology, School of Social and Community Medicine, Bristol, United Kingdom
