# Supplementary material for: Evaluating the Performance of Fine-Mapping Strategies at Common Variant GWAS Loci
Source: PLoS Genet. 2015 Sep 25;11(9):e1005535. doi: 10.1371/journal.pgen.1005535 (PMC4583479; doi:10.1371/journal.pgen.1005535)
Supplement: S1 Table — (PDF) [file pgen.1005535.s007.pdf]

Supplementary Table 1 Fine-mapping results for the twenty six AS loci from Cortes *et al.* using European-only data

Locus	MAF	OR	Most significant ABF variant	Most significant variant log_{10} (ABF)	Most significant variant posterior probability	95% credible set [#]	99% credible set [#]	95% credible locus [bp]	99% credible locus [bp]	95% credible locus coordinates	99% credible locus coordinates	Index variant of primary signal in Cortes et al.	Cortes *et al.* index variant in 99% credible set
ERAP1	0.28	1.38	rs2032890	44.82	0.43	12	12	5,157	5,157	chr5:96121151-96126308	chr5:96121151-96126308	rs30187	Yes
2p15	0.39	1.31	rs4672505	37.57	0.44	4	5	9,241	11,369	chr2:62559204-62568445	chr2:62559204-62570573	rs6759298	Yes
IL23R	0.06	1.68	rs11581607	27.06	0.26	9	11	13,432	17,037	chr1:67699914-67713346	chr1:67699914-67716951	rs11209026	Yes
KIF21B	0.27	1.22	rs296520	16.14	0.20	10	10	7,367	7,367	chr1:200874228-200881595	chr1:200874228-200881595	rs41299637	Yes
21q22	0.25	1.19	rs4817986	12.19	0.13	8	11	3,462	4,361	chr21:40463282-40466744	chr21:40463282-40467643	rs2836883	Yes
RUNX3	0.48	1.15	rs10751775	11.06	0.07	43	48	14,774	16,050	chr1:25291009-25305783	chr1:25289733-25305783	rs6600247	Yes
IL6R	0.4	1.16	rs12133641	10.7	0.11	44	53	90,060	101,676	chr1:154417828-154507888	chr1:154416934-154518610	rs4129267	Yes
FCGR2A	0.49	1.15	rs1801274	10.16	0.44	4	6	10,692	10,692	chr1:161469053-161479745	chr1:161469053-161479745	rs1801274	Yes
GPR35	0.19	1.19	rs34236350	10.07	0.97	1	2	1	1,367	chr2:241568325-241568326	chr2:241568325-241569692	rs4676410	No - Cortes *et al.* variant has r^2 0.9 with rs34236350 but falls outside the 99% credible set and interval
NKX2-3	0.35	1.18	rs11190127	10.04	0.94	5	24	16,954	24,075	chr10:101271981-101288935	chr10:101271788-101295863	rs11190133	Yes
NPEPPS	0.5	1.15	rs4239162	9.95	0.04	113	141	247,336	247,336	chr17:45539116-45786452	chr17:45539116-45786452	rs9901869	Yes
TNFRSF1A	0.39	1.14	rs1800693	8.1	0.62	6	12	9,107	51,070	chr12:6440008-6449115	chr12:6440008-6491078	rs1860545	Yes
IL12B	0.31	1.14	rs6556416	7.61	0.18	12	14	51,413	104,349	chr5:158767332-158818745	chr5:158767332-158871681	rs6556416	Yes
IL27	0.47	1.13	rs34836	7.32	0.05	153	211	412,070	576,700	chr16:28483060-28895130	chr16:28340944-28917644	rs75301646	No - Cortes *et al.* variant not imputed. Within 99% credible interval
TYK2	0.21	1.16	rs74179925	7.07	0.18	21	42	275,321	344,221	chr19:10496620-10771941	chr19:10427720-10771941	rs35164067	Yes
CARD9	0.49	1.12	rs3923827	6.46	0.11	46	58	60,954	61,477	chr9:139240629-139301583	chr9:139240106-139301583	rs1128905	Yes
NOS2	0.38	1.12	rs2779273	6.16	0.04	45	48	37,520	37,520	chr17:26136582-26174102	chr17:26136582-26174102	rs2531875	Yes
ZMIZ1	0.41	1.12	rs1108618	5.9	0.13	19	34	34,451	34,949	chr10:81032531-81066982	chr10:81032531-81067480	rs1250550	Yes
GPR65	0.09	1.19	rs12434101	5.11	0.09	17	22	62,525	76,527	chr14:88426296-88488821	chr14:88426296-88502823	rs11624293	Yes
SH2B3	0.22	1.13	rs11065898	5.1	0.06	146	258	364,621	543,538	chr12:111844217-112208838	chr12:111811603-112355141	rs11065898	Yes
BACH2	0.29	1.11	rs9451298	4.77	0.81	10	13	27,354	27,354	chr6:90645614-90672968	chr6:90645614-90672968	rs17765610	Yes
UBE2L3	0.19	1.16	rs390387	4.56	0.02	93	100	87,614	87,614	chr22:21911219-21998833	chr22:21911219-21998833	rs2283790	Yes
IL7R	0.26	1.12	rs7717955	4.38	0.04	73	150	150,373	340,775	chr5:35803576-35953949	chr5:35644620-35985395	rs11742270	Yes
IL1R1	0.38	1.1	rs4851529	4.16	0.12	295	1,113	819,681	857,929	chr2:102322810-103142491	chr2:102289327-103147256	rs4851529	Yes
UBE2E3	0.43	1.1	rs12620692	4	0.07	45	144	87,466	491,492	chr2:182007799-182095265	chr2:181735286-182226778	rs12615545	Yes
ICOSLG	0.4	1.09	rs2838519	3.24	0.10	36	315	27,772	176,848	chr21:45609109-45636881	chr21:45573200-45750048	rs7282490	Yes
